# Automatic transparency evaluation for open knowledge extraction systems

**DOI:** 10.1186/s13326-023-00293-9

**Published:** 2023-08-31

**Authors:** Maryam Basereh, Annalina Caputo, Rob Brennan

**Affiliations:** 1 School of Computing, Dublin City University, Dublin, Ireland; 2 ADAPT Centre, School of Computer Science, University College Dublin, Dublin, Ireland

**Keywords:** Transparency framework, Automatic transparency evaluation, Open knowledge extraction, FAIRness assessment, Quality evaluation

## Abstract

**Background:**

This paper proposes Cyrus, a new transparency evaluation framework, for Open Knowledge Extraction (OKE) systems. Cyrus is based on the state-of-the-art transparency models and linked data quality assessment dimensions. It brings together a comprehensive view of transparency dimensions for OKE systems. The Cyrus framework is used to evaluate the transparency of three linked datasets, which are built from the same corpus by three state-of-the-art OKE systems. The evaluation is automatically performed using a combination of three state-of-the-art FAIRness (Findability, Accessibility, Interoperability, Reusability) assessment tools and a linked data quality evaluation framework, called Luzzu. This evaluation includes six Cyrus data transparency dimensions for which existing assessment tools could be identified.

OKE systems extract structured knowledge from unstructured or semi-structured text in the form of linked data. These systems are fundamental components of advanced knowledge services. However, due to the lack of a transparency framework for OKE, most OKE systems are not transparent. This means that their processes and outcomes are not understandable and interpretable. A comprehensive framework sheds light on different aspects of transparency, allows comparison between the transparency of different systems by supporting the development of transparency scores, gives insight into the transparency weaknesses of the system, and ways to improve them. Automatic transparency evaluation helps with scalability and facilitates transparency assessment. The transparency problem has been identified as critical by the European Union Trustworthy Artificial Intelligence (AI) guidelines. In this paper, Cyrus provides the first comprehensive view of transparency dimensions for OKE systems by merging the perspectives of the FAccT (Fairness, Accountability, and Transparency), FAIR, and linked data quality research communities.

**Results:**

In Cyrus, data transparency includes ten dimensions which are grouped in two categories. In this paper, six of these dimensions, i.e., provenance, interpretability, understandability, licensing, availability, interlinking have been evaluated automatically for three state-of-the-art OKE systems, using the state-of-the-art metrics and tools. Covid-on-the-Web is identified to have the highest mean transparency.

**Conclusions:**

This is the first research to study the transparency of OKE systems that provides a comprehensive set of transparency dimensions spanning ethics, trustworthy AI, and data quality approaches to transparency. It also demonstrates how to perform automated transparency evaluation that combines existing FAIRness and linked data quality assessment tools for the first time. We show that state-of-the-art OKE systems vary in the transparency of the linked data generated and that these differences can be automatically quantified leading to potential applications in trustworthy AI, compliance, data protection, data governance, and future OKE system design and testing.

## Background

Semantics and linked datasets formalise and classify knowledge in a machine-readable way [1, 2]. This simplifies knowledge extraction, retrieval, and analysis [3, 4]. Open Knowledge Extraction (OKE) is the automatic extraction of structured knowledge from unstructured/semi-structured text and transforming it into linked data [5]. The use of OKE systems as the fundamental component of advanced knowledge services is experiencing rapid growth [6]. However, similar to many modern Artificial Intelligence (AI) based systems, most OKE systems include non-transparent processes.

Transparency is defined as the understandability and interpretability of the processes and outcomes of AI systems for humans [7]. Transparency of AI is needed due to the extensive use of black-box algorithms in modern AI systems [8–14]. Enhancing transparency facilitates scrutability, trust, effectiveness, and efficiency [15]. AI transparency is one of the AI governance main components, which is necessary for accountability [8–10, 15]. Transparency is the single most cited principle in the 84 policy documents reviewed by Jobin et al. [16]. The General Data Protection Regulation (GDPR) also requires transparency by affirming “The right to explanation”, mandating accountability mechanisms and restricting automated decision-making [17].

Automatic transparency evaluation is an important step to enhance the transparency of OKE systems. Automation helps with scalability and saves both time and energy, adding to sustainability. Transparency evaluation allows analysis and indicates effective ways to enhance the transparency of a system under evaluation. Transparency is a multidimensional problem which looks at different aspects of the process, input/s, and output/s of a system, such as their quality, security, and ethics. To the best of our knowledge, to this date, there is no automatic way to evaluate all the transparency dimensions of OKE systems. Accordingly, this paper’s focus is on the automatic transparency evaluation for OKE systems. Our research question is “To what extent can the transparency of OKE systems be evaluated automatically using the state-of-the-art tools and metrics?”. The Cyrus transparency evaluation framework describes a comprehensive set of transparency dimensions, includes a transparency testing methodology, and identifies relevant assessment tools for OKE systems.

The contributions of this paper are as follows: i) the transparency problem for OKE systems is formalised and ii) Cyrus, a new transparency evaluation framework for OKE systems is proposed, iii) state-of-the-art FAIRness assessment [18–20] and linked data quality assessment [21] tools that are capable of evaluating some transparency dimensions are identified and iv) Cyrus and the assessment tools are applied to evaluate the transparency of three state-of-the-art open-source OKE systems by assessing three linked datasets produced from the same corpus [22].

### Open Knowledge Extraction (OKE) systems

OKE is the automatic extraction of structured knowledge from unstructured or semi-structured text and then representing and publishing the knowledge as linked data [5]. OKE usually consist of three main tasks, which are entity and relation extraction, text annotation based on the vocabularies and ontologies, and conversion to RDF^1^ (Resource Description Framework). In this paper, the transparency of three state-of-the-art open-source OKE systems is evaluated. All these systems create Knowledge Graphs (KG) from the same corpus, i.e., Covid-19 Open Research Dataset (CORD-19) [22]. CORD-19 is a corpus of scientific papers on Covid-19 and related historical coronavirus research. An overview of each of these OKE systems is provided in the following paragraphs.

In 2019, Booth et al. [23] created CORD-19-on-FHIR^2^, a linked data version of CORD-19 dataset in FHIR^3^ RDF format. It was produced by data mining the CORD-19 dataset and adding semantic annotations, using the NLP2FHIR pipeline [24] and the FHIR to RDF converter^4^ to create the final linked datasets. The purpose of CORD-19-on-FHIR is to facilitate linkage with other biomedical datasets and enable answering the research question. Currently the entity types of Conditions, Medications and Procedures are extracted using Natural Language Processing (NLP) methods from the titles and abstracts of the CORD-19 dataset. Pubtator^5^ [25] is also used to extract Species, Gene, Disease, Chemical, CellLine, Mutation and Strain.

CORD19-NEKG is another KG construction pipeline for the CORD-19 dataset. It was created by Michel et al. [26]. CORD19-NEKG is an RDF dataset describing named entities in the CORD-19 dataset, which have been extracted using: i) the DBPedia Spotlight [27] named entity extraction tool, which uses DBPedia entities to annotate text automatically; ii) Entity-fishing^6^, which uses Wikidata entities to annotate text automatically; and iii) the NCBO BioPortal Annotator [28], which annotates text automatically with user-selected ontologies and vocabularies.

COVID-KG [29] is another KG based on the CORD-19 dataset. This KG has been built by transforming CORD-19 dataset papers (JSON files and their metadata CSV files) into RDF in two steps: a. Enriching the JSON files using annotations from DBpedia Spotlight, BioPortal Annotator, Crossref API, ORCID API and b. Mapping JSON to RDF using the YARRRML Parser.

### Existing solutions for the transparency of AI models

AI systems have three important components, i.e., 1. Input data or resources, 2. Input transformation process including algorithms and models used, and 3. outputs. For AI to be transparent, each of these components should be transparent. Explainable AI (XAI) aims to turn a non-transparent machine learning model into a mathematically interpretable one. Several studies have suggested using XAI methods to enhance the transparency, however, these methods are often shown to be less accurate than non-transparent algorithms [30–32]. Also, XAI often does not consider whether the explanations are understandable for humans [33–35].

Some researchers suggest auditing or risk assessment [8–10, 36] to increase transparency, which assesses the inputs and outputs of the model assuming the model itself as a black box. However, auditing is the least powerful method among the available methods for understanding black box models’ behaviours [37], since it does not help make the model decision process clear. Logging of algorithm executions can also be helpful by enabling responsible entities to carry out retrospective analysis [38]. Openness of the algorithm’s source code, inputs, and outputs is another way to provide transparency. However, this exposes the system to strategic gaming and does not work for algorithms that change over time and for those with random elements [9].

However, metadata-driven approaches that create a framework to disclose key pieces of information about a model would be more effective in communicating algorithmic performance to the public [15]. Most of the current transparency solutions are metadata-driven [15, 39–41]. Model Cards [42] divide the information about the model into nine groups, i.e., model details (basic information such as model developer/s, model date, version, and type), intended use, factors (demographic or phenotypic groups, environmental conditions, and so on), metrics (e.g., model performance measures or decision thresholds), evaluation data (datasets, motivation, preprocessing), training data, quantitative analyses, ethical considerations, and caveats and recommendations. There are no requirements to reveal sensitive information and organisations only need to disclose basic information about the model.

Inspired by nutrition labels, Yang et al. [43] have suggested a nutrition label for ranking AI systems, as a way to make them transparent. Ranking Facts consist of visual widgets that illustrate details of the ranking methodology or of the output to the users in six groups. These information include the Recipe (describing the ranking algorithm, attributes that matter, and to what extent), the Ingredients (list of the most effective attributes to the outcome), the detailed Recipe and Ingredients widgets (statistics of the attributes in the Recipe and in the Ingredients), the Stability of the algorithm output, the detailed Stability (the slope of the line that is fit to the stability score distribution, at the top-10 and over-all), the Fairness widget (whether the ranked output complies with statistical parity), and the Diversity widget shows diversity with respect to sensitive features.

Another similar approach is FactSheets [44]. In this work, a questionnaire has been created to be filled and published by the stakeholders of AI services. This questionnaire includes 11 sections, i.e., the previous FactSheets filled for the service, a description of the testing done by the service provider, the test results, testing by third parties, Safety, Explainability, Fairness, Concept Drift, Security, Training Data, and Trained Models. Each of the reviewed methods provide a set of information that should be available for their targeted models to be transparent. Table 1 shows differences and commonalities between these approaches.

**Table 1:** Differences and commonalities between AI transparency methods

AI transparency methods	Transparency information												
	Developers	Model date and version	Model type	Intended use	Training data	Evaluation datasets	Evaluation datasets preprocessing	Model performance measures or decision thresholds	Description of model tests	Most effective attributes	Quantitative analyses	Ethical considerations	Limitations
Model Cards [42]	$\checkmark$	$\checkmark$	$\checkmark$	$\checkmark$	$\checkmark$	$\checkmark$	$\checkmark$	$\checkmark$	×	×	$\checkmark$	$\checkmark$	$\checkmark$
Nutritional label for ranking AI systems [43]	×	×	$\checkmark$	×	×	×	×	×	×	$\checkmark$	$\checkmark$	$\checkmark$	×
FactSheets [44]	×	×	$\checkmark$	×	$\checkmark$	×	×	$\checkmark$	$\checkmark$	×	$\checkmark$	$\checkmark$	×

### Existing solutions for data transparency

Similar to solutions for the transparency of AI models, most of the existing solutions for data transparency are metadata-driven. Some of the most significant approaches are overviewed here.

In Datasheets for datasets [45], information about the datasets has been classified in four groups, i.e., composition, collection, preprocessing/cleaning/labelling, and maintenance. Data composition section includes information such as missing information, errors, sources of noise, or redundancies in the dataset. The collection process section contains information such as data validation/verification, mechanisms to collect the data, and validation of the collection mechanisms. The preprocessing/cleaning/labelling section includes information about the raw data and its transformation, e.g., discretisation and tokenisation. Lastly, the maintenance section refers to the information such as the data erratum, applicable limits on the retention of the data, and maintenance of the older versions of the data.

Data Cards method [46] has quite a dynamic format to be applicable to different kinds of data. Information in Data Cards is roughly divided into nine sections, i.e., publishers, licence and access, dataset snapshot-data type, nature of content, known correlations, simple statistics of data features, training, validation, and testing parts, motivation and use-dataset purposes, key domain applications, primary motivations, extended use-safe and unsafe use cases, dataset maintenance, versions, and status, data collection methods, data labelling, and finally fairness indicators.

Data Nutrition Labels [47] consist of seven modules, i.e., metadata, provenance, variables, statistics, pair plots, probabilistic model, and finally ground truth correlations. Metadata module includes information such as filename, format, URL, domain, keywords, dataset size, the number of missing cells, and license. The provenance module contains source and authors’ contact information along with the version history. The variables module provides a textual description of each variable/column in the dataset. The statistics module includes simple statistics for the dataset variables, such as min/max, median, and mean. The pair plots module encompasses histograms and heat maps of distributions and linear correlations between two chosen variables. The probabilistic model module contains histograms and other statistical plots for the synthetic data distribution hypotheses. Lastly, the ground truth correlations module refers to heat maps for linear correlations between a chosen variable in the dataset and variables from the ground truth datasets. One of the interesting contributions in Data Nutrition Labels is visual badges that show information about the dataset. Similar to the AI transparency method, each of the data transparency methods provide a framework of the information that they find necessary for data transparency. Table 2 shows differences and commonalities between these approaches. Inspired by the reviewed model and data transparency methods, we propose a comprehensive transparency evaluation and enhancement framework for OKE systems.

**Table 2:** Differences and commonalities between data transparency methods

Data transparency methods	Transparency information									
	Name	format	URL	Size	data type	publisher	Licence	Version history	Purposes	Features description
Datasheets [45]	×	×	×	×	×	×	×	×	×	×
Data Cards [46]	×	×	×	×	$\checkmark$	$\checkmark$	$\checkmark$	$\checkmark$	$\checkmark$	×
Data Nutrition Labels [47]	$\checkmark$	$\checkmark$	$\checkmark$	$\checkmark$	×	$\checkmark$	$\checkmark$	$\checkmark$	×	$\checkmark$

Data transparency methods	Transparency information								
	Data retention information	Fairness indicators	Collection mechanism	Validation and verification	Errors, noise, redundancies	Missing values	Maintenance	Preprocessing, cleaning, labelling	Dataset statistics
Datasheets [45]	$\checkmark$	×	$\checkmark$	$\checkmark$	$\checkmark$	$\checkmark$	$\checkmark$	$\checkmark$	×
Data Cards [46]	×	$\checkmark$	$\checkmark$	×	×	×	$\checkmark$	$\checkmark$	$\checkmark$
Data Nutrition Labels [47]	×	×	×	×	×	$\checkmark$	×	×	$\checkmark$

### Existing solutions for the evaluation of AI systems’ transparency

To the best of our knowledge, there are no automatic methods that cover the evaluation of all the transparency dimensions for AI systems. However, there are some checklists to measure fairness, accountability, and transparency of AI systems, regardless of the techniques that are used in building systems. Shin [7] uses a 27 measurements checklist on a 7-point scale for seven criteria, i.e., fairness, accountability, transparency, explainability, usefulness, convenience, and satisfaction, to evaluate user perceptions of algorithmic decisions. However, the checklist itself is not publicly available. In another work, Shin et al. [48] have proposed a survey with transparency among its variables. However, it cannot be independently used and needs other approaches to measure these criteria. Jalali et al. [49] evaluated the transparency of reports for 29 COVID-19 models using 27 Boolean criteria. These criteria have been adopted from three transparency checklists [50–52] which include reproducibility and transparency indicators for scientific papers and reports. Jalali et al.’s transparency assessment checklist was used in [53] for the transparency evaluation.

### Automatic transparency evaluation

Quality and transparency are entangled concepts [12, 15]. In 2012, Zaveri et al. [54] proposed a comprehensive linked data quality evaluation framework, consisting of six quality categories and 23 quality dimensions, for each dimension a number of metrics has been identified in the literature. Based on the Data Quality Vocabulary^7^, a category “Represents a group of quality dimensions in which a common type of information is used as quality indicator.” and a dimension “Represents criteria relevant for assessing quality. Each quality dimension must have one or more metric to measure it”. A number of quality evaluation metrics have been implemented in open source linked data quality evaluation tools, such as the two following tools: RDFUnit [55] and Luzzu[21].

In [55], inspired by test-driven software development, a methodology has been proposed for linked data quality assessment based on SPARQL query templates, which are then instantiated into concrete quality test queries. Through this approach, domain specific semantics can be encoded in the data quality test cases, which allows the discovery of data quality problems beyond conventional methods. An open access tool, named RDFUnit^8^ has been built based on this method.

Debattista et al. [21] propose Luzzu, a conceptual methodology for assessing linked datasets and a framework for linked data quality assessment. Luzzu allows defining new quality metrics, creating RDF quality metadata and quality problem reports, provides scalable dataset processors for data dumps, SPARQL endpoints, and big data infrastructures, and a customisable ranking algorithm for user-defined weights. Luzzu scales linearly against the number of triples in a dataset. Luzzu is open-source and has 29 quality evaluation metrics already implemented.

In addition to the above, our prior work has shown that FAIR principles [56] can be used to evaluate some transparency dimensions [57]. FAIR principles are well-accepted data governance principles, which have originally been proposed to enhance usability of scholarly digital resources for humans and machines [58, 59]. FAIR principles include four criteria for findability, two for accessibility, three for interoperability, and one (including three sub-criteria) for reusability. Since their emergence in 2016, several automatic tools [18–20] have been suggested to check if digital objects (resources, datasets) are aligned with the FAIR principles.

## Methods

This section introduces a new transparency evaluation framework for OKE systems called Cyrus. We also identify a set of automatic linked data quality evaluation tools and methods, which can be used to evaluate some transparency dimensions for KGs, as outputs of OKE systems. Finally, we describe an experiment in which the framework is used to evaluate the transparency of the three KGs that are the outputs of three state-of-the-art OKE systems [23, 26, 29]. All of these three OKE systems have been built to generate KGs from the same corpus, i.e., CORD-19.

### Cyrus: a transparency evaluation framework for OKE systems

As it can be seen in Table 1, Table 2, and Table 3 different methods provide different sets of information for AI and data transparency and introduce different categorisation for transparency information. None of these methods are comprehensive. For example, Datasheets introduce more technical and statistical information, while Data Cards and Data Nutrition Labels focus on different data provenance aspects. Moreover, none of the reviewed methods particularly introduce transparency information for OKE systems and KGs. Accordingly, we propose a transparency evaluation framework for OKE systems, called Cyrus. Similar to other AI systems, OKE systems have three main components, i.e., input data and resources, input transformation process including algorithms and models used, and the outputs. In our model of transparency, the transparency of an OKE depends on the transparency of its components. Accordingly, if there is enough metadata about the components of an OKE system, that system is itself transparent. Therefore, Cyrus consists of

1. A comprehensive list of data transparency dimensions and attributes for the input (unstructured or semi-structured text) and output (knowledge graphs) and resources (ontologies and vocabularies) of the OKE systems
2. And a list of transparency dimensions and attributes for the input transformation process^9^ that is done within the OKE.

A full transparency evaluation of an OKE system can be done by evaluating its input, output, resources, and input transformation processes (algorithms and models) against Cyrus.

Quality and transparency are closely connected [12, 15]. Accordingly, the data transparency in Cyrus has been created based on the state-of-the-art data transparency methods [45, 47] mapped to the Zaveri et al.’s conceptual model of linked data quality metrics [54]. We extended five of Zaveri et al.’s linked data quality dimensions’ attributes, i.e., understandability, accuracy, conciseness, volatility, and completeness for the requirements of data transparency. In addition, while Zaveri et al. introduce provenance as a metric in the believability dimension, due to the importance of the provenance information for transparency and the amount of information it covers, we propose provenance as an separate dimension for data transparency. Provenance information “describes the origins and the history of data in its life cycle” [60]. It is a crucial component of workflow systems that helps their reproducibility [61]. In addition, an important part of transparency is information about ethics, privacy, and security, such as if data is confidential, if data collection/generation has gone through an ethics committee review, and if mechanisms have been provided to secure private/confidential data. As a result, data transparency in Cyrus consists of two categories, i.e., “quality” and “security and ethics”. The quality category consists of 24 dimensions, 23 of which introduced by Zaveri et al. plus provenance. Security and ethics category includes four dimensions, i.e., security and privacy, disclosure and data provisioning, laws and policies, and ethical. Table 3 shows the transparency framework categories, dimensions, and their attributes.

**Table 3:** Cyrus - Data transparency framework

Categories	Dimensions	Attributes
Quality	Provenance	1. An access point to the raw data [45]
		2. The context - timeframe of data collection [45, 47]
		3. The context - location of data collection [62]
		4. The data collection agent (applications, sensors, human users) [62]
		5. The number of participants, the eligibility criteria, the follow-up times, if there has been participants [63, 64]
		6. Source data transformation/preprocessing [15, 45]
		7. Data assumptions [15, 49, 62]
		8. An access point to the model and algorithm transparency information, if the data/resource is an output of an automatic/semi-automatic process
		9. Intended uses of the data [62, 65]
		10. Intended data users [62, 65]
		11. Data usage history, including applications that have processed the data and the purpose of the use [62, 65]
		12. A contact point [62, 66]
		13. Citation details [42]
		14. Sources of funding [47]
		15. Dataset version history
	Understandability	1. Correlations between different dataset properties [47]
		2. The stratifications into ordinal, nominal, continuous, and discrete, e.g., least/most entries, min/max, median, mean [47]
	Accuracy	1. Sources of errors and noise in the dataset [45]
		2. An access point to the erratum, if applicable [45]
	Conciseness	1. Sources of redundancies in the dataset [45]
	Volatility	1. Dates of planned updates of the dataset [45]
		2. Mechanisms for distributing updates [45]
		3. Mechanisms to support/host/maintain the older versions of the dataset, if applicable [45]
		4. Mechanisms to communicate/distribute the obsolescence of the older versions of the dataset, if applicable [45]
		5. Mechanisms to extend/augment/build on/contribute to the dataset [45]
		6. Validation/verification procedures for dataset extensions [45]
		7. Mechanisms for communicating/distributing dataset extensions [45]
	Completeness	1. Data limitations, e.g., missing information [15, 45, 47, 49, 62]
Security and ethics	Security and privacy	1. Existence of personal or confidential data [15, 45, 47, 66]
		2. Security and privacy management techniques for the data [15, 45, 66]
		3. Information about breaches of data over a period of time [62, 65]
	Disclosure and Data Provisioning (for data transmitted across organisations)	1. Contracts and legal agreements concerning the data disclosure [62, 65]
		2. Limits on the retention of the data, if applicable [45]
		3. Financial agreements [62, 65]
		4. Technical mechanisms used for data transmission [62, 65]
	Laws and policies [45, 62, 65]	1. Availability of laws, regulations, and organisational policies associated with the data of interest to all subjects
	Ethical	1. Ethical review [45, 47, 62]
		2. Informed consent for data collection [45, 47, 62, 66]

In Cyrus, input transformation process transparency consists of provenance, process, review, and security and ethics dimensions. See the appendix for the full list.

### Experiment

In this section, we describe an experiment that was conducted to evaluate the transparency of OKE systems using the state-of-the-art tools and metrics. Our goal is to find the transparency weaknesses of three state-of-the-art OKE systems with the same input corpus and show the extent to which the transparency of OKE systems can be automatically evaluated using state-of-the-art tools and metrics. It is worth mentioning that this evaluation includes six transparency dimensions that currently can be evaluated using the existing automatic tools and metrics.

#### Hypothesis

Our hypothesis is that Luzzu and FAIRness assessment tools can identify transparency weaknesses in OKE systems.

#### Input dataset

CORD-19 [22] is the input dataset for the OKE systems. This dataset is a corpus of scientific papers on Covid-19 and related historical coronavirus research, which includes 18.7 GB of harmonised and deduplicated papers from the World Health Organisation, PubMed Central, bioRxiv, and medRxiv. The final version of CORD-19 was released on June 2, 2022.

#### Experimental setup

**Fig. 1:**
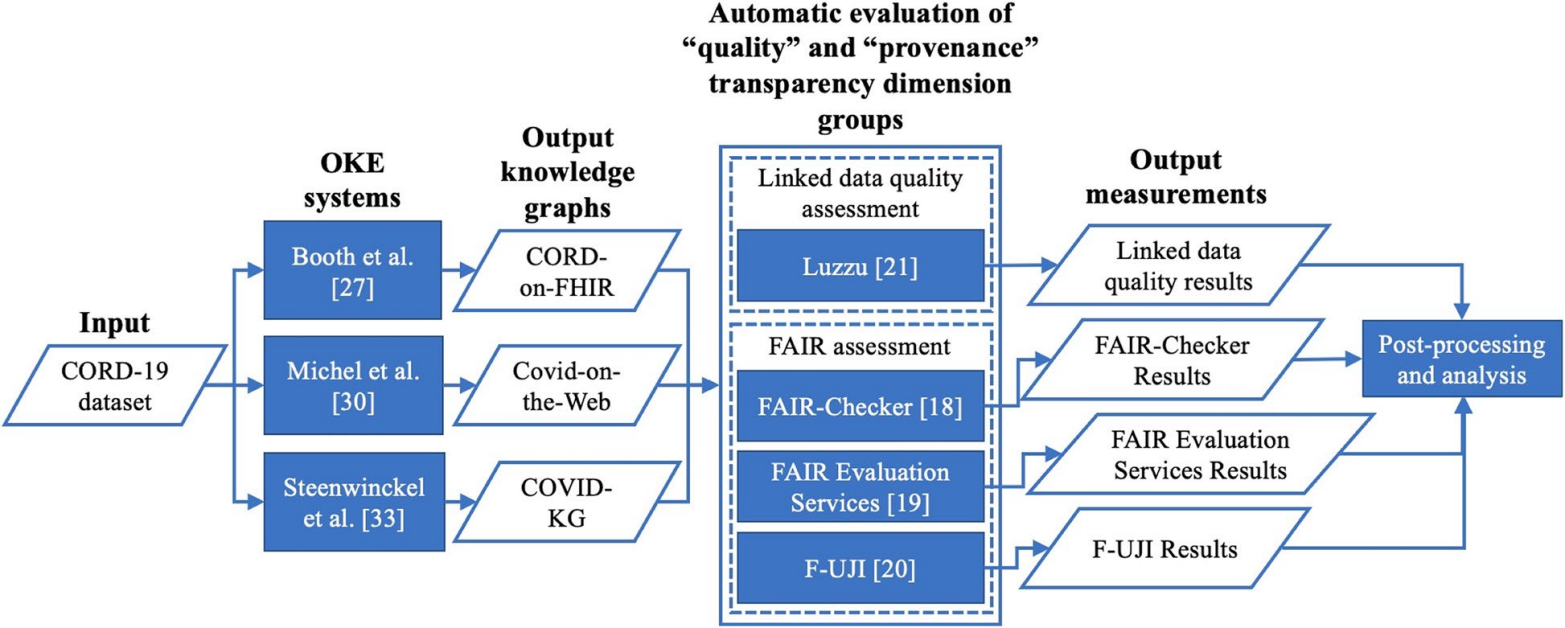
Experimental setup to automatically identify transparency weaknesses in Open Knowledge Extraction (OKE) systems

The experiment setup is illustrated in Fig. 1.

As shown in Fig. 1, first, three state-of-the-art OKE systems automatically construct three KGs from CORD-19 dataset. Second, Luzzu and three automatic FAIRness assessment tools, i.e., FAIR-Checker, FAIR Evaluation Services, and F-UJI are used to evaluate six transparency dimensions, i.e., provenance, interpretability, understandability, licensing, availability, and interlinking for the output KGs. Finally, as the post-processing, the mean transparency and the mean results for each of the transparency dimensions are calculated for each of the KGs, which then will be compared. In this experiment, Luzzu (v4.0), FAIR-Checker (v1.0.4), FAIR Evaluation Services (latest release: July 2018), and F-UJI (v1.0.0) have been used. At the time of conducting the experiment (November 2021), the available automatic FAIRness assessment tools^10^ were tested and FAIR-Checker (v1.0.4), FAIR Evaluation Services (latest release: July 2018), and F-UJI (v1.0.0) were the only automatic FAIRness assessment tools that were functioning properly. Table 4 illustrates the components of the three state-of-the-art OKE systems that are evaluated in the experiment.

**Table 4:** The structure of the three state-of-the-art OKE systems used in the experiment

OKE systems	OKE systems’ components		
	Entity and relation extraction	Resources	Conversion to RDF
Booth et al. [23]	NLP2FHIR + PubTator	COVID-19 PICO Ontology	RDF Convertor
Michel et al. [26]	DBpedia Spotlight + BioPortal Annotator + Entity-fishing	DCMI, Bibliographic, FOAF, Schema.org, WAV, and PROV-O	Morph-xR2RML
Steenwinckel et al. [29]	DBpedia Spotlight + BioPortal Annotator + Crossref API + ORCID	COVID 19, CIDO, FLU, ans FaBio	YARRRML Parser

Each of these OKE systems create different KGs by extracting different entity types and relations. CORD-19-on-FHIR, includes Conditions, Medications, Procedures, Species, Gene, Disease, Chemical, CellLine, Mutation and Strain entity types. CORD19-on-the-Web includes DBpedia, Wikidata, user-selected ontologies and vocabularies’ named entities that are present in the CORD-19 dataset. COVID-KG has been built by transforming CORD-19 dataset papers (JSON files) into RDF. These OKE systems have been chosen for this experiment, because all of them use CORD-19 as their input, are open-source, and their output KGs are openly available. For this experiment, the available output KGs of these three OKE systems have been downloaded and as a sample, one part of the three KGs relevant to one of the papers has been chosen accidentally and used.

#### Experiment measurements

As mentioned in the experiment setup, Luzzu and three FAIRness assessment tools, i.e., FAIR-Checker, FAIR Evaluation Services, and F-UJI are used as the evaluation tools, in this experiment. 38 linked data quality evaluation metrics have been implemented in Luzzu v4.0. We classified them into core, middle, and supportive classes according to their importance and used the core ones for this experiment. Table 5 illustrates related FAIRness assessment tools and Luzzu metrics and Cyrus data transparency dimensions they are related to.

**Table 5:** FAIR and Linked data quality evaluation metrics used in the experiment

Dimensions	Metrics
Provenance	Basic Provenance - Luzzu
	Extended Provenance - Luzzu
	Reusability - FAIR tools
Interpretability	No Blank Node Usage - Luzzu
	Undefined Classes and Properties - Luzzu
Understandability	Vocabulary Usage Indication - Luzzu
	Human Readable Labelling and Description - Luzzu
	Presence of URI RegEx - Luzzu
Licensing	Human Readable License - Luzzu
	Machine Readable License -Luzzu
Availability	Findability - FAIR tools
	Accessibility - FAIR tools
Interlinking	Links to External Data Providers - Luzzu
	Estimated Links to External Data Providers - Luzzu
	Interoperability - FAIR tools

In Luzzu, two provenance metrics have been implemented, the basic provenance metric and the extended provenance metric (See Table 5). The basic one measures if a dataset has the most basic provenance information, which is information about the creator or publisher of the dataset. It means that each dataset should include either dc:creator or dc:publisher properties, as a minimum requirement. The extended provenance metric checks if a dataset has the required provenance information that would enable the consumer to know the origin (where), the owner (who), and the activity that creates the triple (how). In this metric, the following requirements are considered.

- Identification of an Agent;
- Identification of Activities in an Entity;
- Identification of a Data source in an Activity;
- Identification of an Agent for an Activity

Accordingly, the existence of PROV:wasAttributedTo, PROV:wasGeneratedBy, PROV:wasUsed, PROV:wasAssociatedWith, PROV:Entity, and PROV:Activity properties are checked in the dataset’s metadata. Notice that these information should exist through these properties, i.e., in specific format to be counted by Luzzu. In comparison to Luzzu, FAIR criteria evaluates not only the publisher/owner and licensing information of the resource, but also title, size, link, data definition and/or properties and data format, and data versions history.

All the metrics are double type variables, except the following. The interlinking metrics are integer variables, counting the links to external data providers. The human readable license, machine readable license, and the presence of URI RegEx metrics are nominal variables and their values can be either true or false. For normalisation purposes, the false and the true values will be considered as zeros and ones, respectively. Next, we discuss the results.

## Results

Luzzu results are shown in Table 6. The experiment was run on a computer with an Quad-Core Intel Core i7 processor running at 1.2 GHz using 16 GB of RAM, running macOS Big Sur version 11.6.7.

**Table 6:** Luzzu results for the evaluation of provenance and quality categories

Linked data quality dimensions [54]	Metrics relevant to transparency [54]	Luzzu results for each of the KGs		
		CORD-19-on-FHIR	Covid-on-the-Web	COVID-KG
Licensing	Human Readable License	false	false	false
	Machine Readable License	false	false	false
Interlinking	Links to External Data Providers	0	0	0
	Estimated Links to External Data Providers	0	0	0
Interpretability	No Blank Node Usage	0.179	1.0	1.0
	Undefined Classes and Properties	0.085	0.749	0.573
Understandability	Human Readable Labelling and Description	0.0	0.0	0.033
	Vocabulary Usage Indication	0.0	0.0	0.0
	Presence of URI RegEx	false	false	false
Provenance	Extended Provenance Metric	0.0	0.0	0.0
	Basic Provenance Metric	0.0	1.0	0.0

According to the results

- All three KGs do not have machine-readable and human-readable licensing information in their metadata
- All three KGs are not linked to external data providers (interlinking = 0)
- In terms of interpretability
  - Covid-on-the-Web and COVID-KG have no blank nodes (No Blank Node Usage = 1.0) and CORD-19-on-FHIR has blank nodes (No Blank Node Usage $< 1.0$)
  - CORD-19-on-FHIR has the highest and Covid-on-the-Web has the lowest number of undefined classes and properties. The more the value is close to zero the more the number of undefined classes and properties
- In terms of understandability
  - CORD-19-on-FHIR and Covid-on-the-Web do not contain human readable labelling and descriptions (The RDF files do not contain rdfs:label and rdfs:comment properties)
  - Vocabularies used in them are not indicated in their metadata, and they do not contain regular expressions for their URIs)
- In terms of provenance
  - None of the KGs include extended provenance information (PROV:wasAttributedTo, PROV:wasGeneratedBy, PROV:wasUsed, PROV:wasAssociatedWith, PROV:Entity, and PROV:Activity properties do not exist in the RDF files)
  - Only Covid-on-the-Web includes basic provenance information (dc:creator or dc:publisher properties exist in the RDF file)

FAIR evaluation results calculated by FAIR-Checker, FAIR Evaluation Services, and F-UJI have been mentioned in Tables 7, 8, and 9, respectively.

**Table 7:** FAIR-Checker FAIR evaluation results for the output KGs of three state-of-the-art OKE systems

FAIR	FAIR metrics	FAIR results for each of the KGs		
		CORD-19-on-FHIR	Covid-on-the-Web	COVID-KG
Findability	F1A- Unique IDs	2	2	2
	F1B- Persistent IDs	0	0	2
	F2A- Structured metadata	0	2	2
	F2B- Shared vocabs for metadata	0	1	1
Accessibility	A1.1- Open resolution protocol for data	2	2	2
Interoperability	I1- Metadata Knowledge Representation Language	0	2	2
	I2- Metadata uses semantic resources	0	1	1
	I3- Metadata contains qualified outward references	0	0	2
Reusability	R1.1- Metadata includes license	0	0	2
	R1.2- Metadata includes provenance	0	0	0
	R1.3- Metadata follows a standard recommended by the target research community	0	1	1

**Table 8:** FAIR Evaluation Services results for the output KGs of three state-of-the-art OKE systems

FAIR	FAIR metrics	FAIR results for each of the KGs		
		CORD-19-on-FHIR	Covid-on-the-Web	COVID-KG
Findability	F1A- Unique IDs	1	1	Error
	F1B- Persistent IDs	0	0	Error
	F1C- Persistent Data IDs	0	0	Error
	F2A- Structured metadata	0	1	Error
	F2B- Shared vocabs for metadata	0	1	Error
	F3- Data ID in Metadata	0	0	Error
	F3B- Metadata ID in Metadata	0	0	Error
	F4- Searchable in major search engines	0	0	Error
Accessibility	A1: Metadata contains machine-readable DAL and DAC	0	0	0
	A1.1- Open resolution protocol for data	0	0	1
	A1.1- Open resolution protocol for metadata	1	1	1
	A1.2- Data authentication and authorisation	0	0	1
	A1.2- Metadata authentication and authorisation	1	1	1
Interoperability	I1- Metadata Knowledge Representation Language-Weak	0	1	1
	I1- Metadata Knowledge Representation Language-Strong	0	1	1
	I1- Data Knowledge Representation Language-Weak	0	0	0
	I1- Data Knowledge Representation Language-Strong	0	0	0
	I2- Metadata uses semantic resources-Weak	0	1	1
	I2- Metadata uses semantic resources-Strong	0	0	0
	I3- Metadata contains qualified outward references	0	1	1
Reusability	R1.1- Metadata includes license-Weak	0	0	0
	R1.1- Metadata includes license-Strong	0	0	0

**Table 9:** F-UJI FAIR evaluation results for the output KGs of three state-of-the-art OKE systems

FAIR	FAIR metrics	FAIR results for each of the KGs		
		CORD-19-on-FHIR	Covid-on-the-Web	COVID-KG
Findability	F1A- Unique IDs	1	1	1
	F1C- Persistent Data IDs	0	0	0
	F2- Structured metadata and shared vocabs for metadata	0	0.5	0.5
	F3- Data ID in Metadata	0	0	1
	F4- Searchable in major search engines	0	0	1
Accessibility	A1: Metadata contains machine-readable DAL and DAC	0	0	0.5
	A1.1- Open resolution protocol for data	0	0	1
	A1.1- Open resolution protocol for metadata	0	1	1
Interoperability	I1- Metadata Knowledge Representation Language	0	1	1
	I2- Metadata uses semantic resources	0	1	0
	I3- Metadata contains qualified outward references	0	1	1
Reusability	R1- Metadata specifies the content of the data	0	0.5	1.5
	R1.1- Metadata includes license	0	0	0.5
	R1.2- Metadata includes provenance	0	2	1.5
	R1.3- Metadata follows a standard recommended by the target research community	0	0.5	0.5
	R1.3- Data is available in a file format recommended by the target research community	0	0	0

Based on the results, findability results almost 100% match for those criteria which are common between at least two of the tools. Accessibility results almost 66.7% match for those criteria which are common between at least two of the tools. Interoperability results almost 77.8% match for the criteria which are common between at least two of the tools. Reusability results almost 66.7% match for the metrics which are common between at least two of the tools. In most cases, FAIR-Checker has been inconsistent with the other tools. Table 10 shows normalised mean of the FAIR results for the output KGs of three state-of-the-art OKE systems.

**Table 10:** Normalised mean of the FAIR results for the output KGs of three state-of-the-art OKE systems

Linked datasets	FAIR-Checker				FAIR Evaluation Services				F-UJI			
	F	A	I	R	F	A	I	R	F	A	I	R
CORD-19-on-FHIR	0.25	1	0	0	0.125	0.4	0	0	0.143	0	0	0
Covid-on-the-Web	0.75	1	0.667	0.333	0.375	0.4	0.571	0	0.214	0.333	0.75	0.3
COVID-KG	1	1	1	0.667	Error^a^	0.8	0.571	0	0.643	1	0.5	0.4

^a^The FAIR Evaluation Services tool returns “server error’ for COVID-KG findability

Based on FAIR-Checker and F-UJI results, COVID-on-FHIR, Covid-on-the-Web, and COVID-KG respectively scored the highest to the lowest for findability. FAIR Evaluation Services results also shows the same order for COVID-on-FHIR and Covid-on-the-Web. However, it returns “Server Error” for COVID-KG’s findability. FAIR-Checker uses one metric to evaluate accessibility and based on that all KGs have equal accessibility. However, based on FAIR Evaluation Services and F-UJI COVID-KG has the highest accessibility. Based on the results, all three tools have scored zero for the interoperability of CORD-19-on-FHIR but they do not have common orders for the other KGs’ Interoperability. All the tools have scored zero for the reusability of CORD-on-FHIR. COVID-KG, Covid-on-the-Web, and CORD-19-on-FHIR have respectively the highest to the lowest reusability, based on FAIR-Checker and F-UJI results.

As it can be seen in Tables 7, 8, and 9, the three FAIRness assessment tools used in the experiment, use different metrics to evaluate FAIR. FAIR Evaluation Services tool has more in-depth metrics for findability, accessibility, and interoperability and F-UJI metrics are more well-formed for reusability. Accordingly, in the following tables, the FAIR results are aggregated by using FAIR Evaluation Services results for findability, accessibility, and interoperability and F-UJI results for reusability^11^.

Table 11 shows the mean transparency of each of the OKE output KGs, sparated by transparency dimensions. Based on the results, Covid-on-the-Web scored the highest for interperetability and provenance and COVID-KG scored the highest for availability.

**Table 11:** Mean transparency results separated by transparency dimensions

Linked datasets	Transparency dimensions					
	Licensing	Interlinking	Interpretability	Understandability	Provenance	Availability
CORD-19-on-FHIR	0	0	0.132	0.0	0.0	0.263
Covid-on-the-Web	0	0.19	0.875	0.0	0.43	0.387
COVID-KG	0	0.19	0.787	0.01	0.13	0.72

**Fig. 2:**
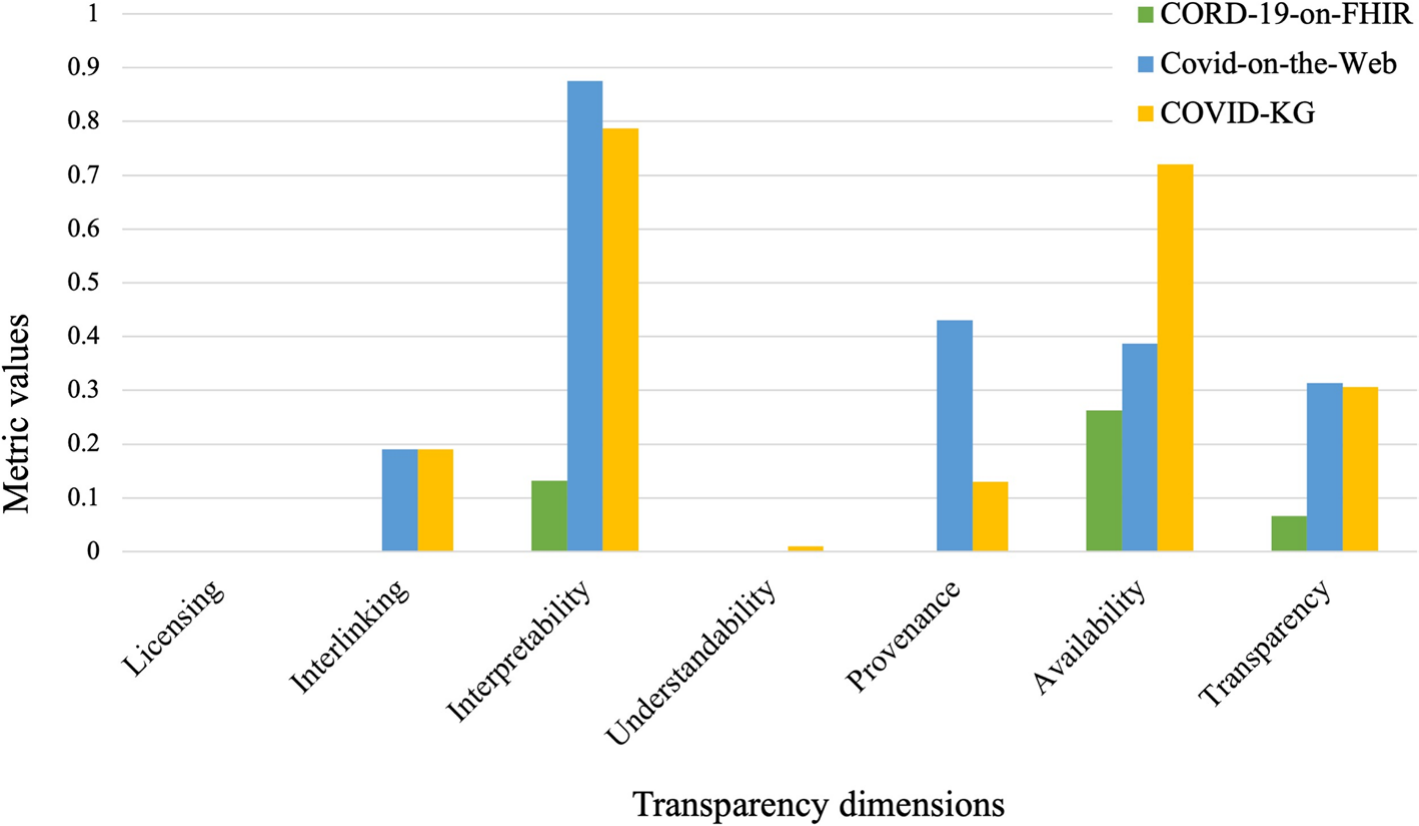
Mean transparency results for each of the three KGs

The transparency results can be compared in Fig. 2. Based on the results, Covid-on-the-Web has the highest mean transparency, slightly higher than COVID-KG.

## Discussion

Notice that results are only related to those transparency dimensions that can be currently evaluated automatically and do not include all the related information presented in the proposed framework. Based on the results, FAIRness assessment tools and Luzzu are capable of evaluating some quality and provenance information for OKE systems, which means these tools can show transparency weaknesses of OKE systems. Accordingly, our hypothesis is approved. This means that using these tools allows effective transparency enhancement by showing the points that need improvement. This has potential applications in trustworthy AI, compliance, data protection, data governance, and future OKE system design and testing.

## Conclusion

This paper answers the research question i.e., “To what extent can the transparency of OKE systems be evaluated automatically using the state-of-the-art tools and metrics?” through

- Proposing Cyrus, a comprehensive transparency framework which includes the metadata that is needed to both assess and enhance the transparency of OKE systems (Framework section). This helps with identifying gaps in automatic transparency evaluation of OKE systems and has been an initial step for creating a transparency catalogue for OKE systems;
- Automatically evaluating six transparency dimensions, i.e., provenance, interpretability, understandability, licensing, availability, and interlinking for the output of three state-of-the-art OKE systems (Table 4), using three automatic FAIRness assessment tools i.e., FAIR-Checker, FAIR Evaluation Services, and F-UJI and Luzzu (Results section). The results (Table 11 and Fig. 2) show that FAIRness assessment tools and some linked data quality evaluation metrics can show transparency weaknesses of the OKE systems.

There are limitations in our experiment, as follows. Small parts of the three KGs have been evaluated using the Luzzu tool. The scores are coming from those transparency dimensions that can be currently evaluated using the state-of-the-art tools and do not include all dimensions presented in the framework. Also, the quality and provenance weaknesses of the outputs of the three state-of-the-art OKE systems are only applicable to these systems and may not be generalised.

In the future, we plan to create a transparency catalogue (specification) based on Cyrus, which gives a standard format including needed ontologies and vocabularies that allows recording the transparency information in a standard machine-readable way. We also plan to expand the automatic transparency evaluation for OKE systems by creating more tools and metrics, based on our proposed framework, Cyrus.

## Data Availability

The input dataset for the three OKE systems, analysed in the current study is available in the Allen Institute for AI repository, https://ai2-semanticscholar-cord-19.s3-us-west-2.amazonaws.com/historical_releases.html. [22]. The three state-of-the-art OKE systems, analysed in the current study are available at: $\bullet$ CORD-19-on-FHIR: Available from https://github.com/fhircat/CORD-19-on-FHIR$\bullet$ Covid-on-the-Web: Available from https://github.com/Wimmics/covidontheweb/dataset$\bullet$ COVID-KG: Available from https://github.com/GillesVandewiele/COVID-KG The linked data quality evaluation framework, Luzzu, is available from https://github.com/Luzzu. F-UJI FAIRness assessment tool is available from https://github.com/pangaea-data-publisher/fuji. FAIR-Checker FAIRness assessment tool is available from https://github.com/IFB-ElixirFr/fair-checker. FAIR Evaluation Services tool’s code is not publicly available but the tool is available from https://fairsharing.github.io/FAIR-Evaluator-FrontEnd/#!/ for use and for adding more tests.

## Appendix: The full list of input transformation process transparency categories, dimensions, and attributes

**Table 12:** Cyrus - Input transformation process transparency framework

Categories	Dimensions
Model provenance information	1. Model title
	2. A link or other access point to the Model
	3. Implementation information
	$\bullet$Software/s used [15, 49]
	$\bullet$Codes [49, 67]
	$\bullet$Documentation for the codes [49]
	$\bullet$If and when an algorithm is being employed [15]
	4. High-level visualisation [49]
	5. Model use
	$\bullet$Primary intended use cases [42, 44, 68]
	$\bullet$Out-of-scope use cases [42]
	$\bullet$Primary intended users [42, 44, 49, 66]
	$\bullet$Information on how to use the system [42, 66]
	6. Model limitations [49, 63]
	7. Paper or other resource for more information [42, 44]
	8. Licensing information [42, 66]
	9. The stakeholders^a^ [15, 38, 42, 44, 66]
	10. A contact point [42, 66]
	11. Model versions and dates [42, 44]
	12. Metadata dates and versions [44]
	13. Citation details [42]
	14. Sources of funding [49, 63]
Modelling information	1. Modelling method [42–44, 49, 62, 63, 65]
	2. Information about the model output/s
	$\bullet$Model output/s (Model questions) [49, 63]
	$\bullet$A link or other access point to the model output/s
	$\bullet$A link or other access point to the outputs’ transparency information
	3. Training dataset/s [15, 42, 44, 62, 67]
	$\bullet$List of the training datasets [44]
	$\bullet$Training dataset transparency information [44]
	$\bullet$Dataset size information
	$\bullet$Sample size [44, 63, 64]
	$\bullet$Rationale for the sample size [63]
	$\bullet$Preprocessing techniques used [42]
	4. Information about model input/s
	$\bullet$Input data
	$\bullet$Input data transparency information [44]
	$\bullet$Dataset size information: Sample size [44, 63, 64], Rationale for the sample size [63]
	$\bullet$Preprocessing techniques used [42]
	$\bullet$Features or variables used in the algorithm [15]
	$\bullet$Feature weights or regression coefficients [15, 43, 49, 63]
	$\bullet$Modelling assumptions [15, 49]
	$\bullet$Statistical analysis methods for attributes^b^ [42, 43, 63]
	$\bullet$Other resources
	$\bullet$List of other resources used as inputs for the system
	$\bullet$Links or other access points to the provenance information for each of the resources
	5. Information about model parameters
	$\bullet$Model parameters and values[42, 49]
	$\bullet$Model calibration (parameter estimation) [42, 49]
	6. Model updates or adjustments [63, 64]
	$\bullet$Any model updates or adjustments^c^ arising from the validation
	$\bullet$Results from any model updating^d^
	7. Model evaluation [15, 42, 44, 49, 64]
	$\bullet$Evaluation dataset/s [42, 44, 67]
	$\bullet$Test and holdout data transparency information [44]
	$\bullet$Dataset size information: Sample size [44, 63, 64], Rationale for the sample size [63]
	$\bullet$Preprocessing techniques used [42]
	$\bullet$Comparison between validation and development datasets [63, 64]
	$\bullet$Methods used to evaluate model performance, e.g., cross validation [64]
	$\bullet$Performance measures results [15, 42–44, 49, 63, 64, 69]
	$\bullet$Rationale for performance measures [44]
	$\bullet$Benchmarking against standard datasets [44, 49]
	$\bullet$Reliability analysis, e.g., baseline survival [64]
	$\bullet$FAIR [57]
	$\bullet$Third party performance verifications [44]
	$\bullet$Concept drift [44]
	$\bullet$Interpretation of results [63, 64]
	$\bullet$If objectives are met considering the results
	$\bullet$Model limitations
	$\bullet$Model generalisability
	$\bullet$Sources of errors
	8. Model Explainability [42, 44, 62, 65, 66]
	$\bullet$Explainability/interpretability approaches used [42, 44, 62, 66]
	$\bullet$The target user of the explanation [44]
	$\bullet$Any human validation of the explainability of the algorithms [44]
Ethical information (bias/unfairness, privacy, security, risk related factors, and conformance of the model/system)	1. Personal information
	$\bullet$Personal information used [15]
	$\bullet$Informed consent
	$\bullet$Information consent form
	$\bullet$Options to withdraw personal data [44]
	$\bullet$The withdrawal procedure [44]
	2. Information about possible adverse outcomes [15, 38, 42–44, 62, 66, 68]
	$\bullet$An analysis of possible adverse outcomes [63]
	$\bullet$Impact of possible adverse outcomes
	$\bullet$Unfairness and bias analysis
	$\bullet$Sources of bias or unfairness [44]
	$\bullet$Bias/Unfairness measures
	$\bullet$Remediation for possible adverse outcomes [38, 44, 68]
	$\bullet$Remediation procedures
	$\bullet$value of bias estimates before and after remediation
	$\bullet$Performance metrics changes after remediation
	3. Privacy and security management information [42, 44, 62]
	$\bullet$Possible privacy and security weaknesses, i.e., ways the system can be attacked or abused
	$\bullet$Privacy and security management approaches, e.g., ways to handle potential security breaches
	4. Information about the filtered elements of a curated experience [15]
	5. Potential conflicts of interests [49]
Model review information	1. Plan for continuous monitoring [15, 38, 44, 68]
	2. Retrospective analysis of disasters [38]

^a^The owner/s, person, or organisation developing the model

^b^Such as min, max, and median values at the top-10 and over-all

^c^Such as recalibration, recalibration, predictor effects adjusted, or new predictors added

^d^i.e., model specification, model performance

## Abbreviations

OKE: Open Knowledge Extraction
CORD-19: COVID-19 Open Research Dataset
FAIR: Findable, Accessible, Interoperable, Reusable
AI: Artificial Intelligence
FAccT: Fairness, Accountability, and Transparency
GDPR: General Data Protection Regulation
RDF: Resource Description Framework
KG: Knowledge Graph
FHIR: Fast Healthcare Interoperability Resources
NLP: Natural Language Processing
XAI: Explainable AI

## Footnotes

1 Is the W3C standard data model for description and exchange of graph data on the web

2 https://github.com/fhircat/CORD-19-on-FHIR

3 Fast Healthcare Interoperability Resources

4 https://github.com/BD2KOnFHIR/fhirtordf

5 https://www.ncbi.nlm.nih.gov/research/pubtator/

6 https://github.com/kermitt2/entity-fishing

7 https://www.w3.org/TR/vocab-dqv/

8 https://github.com/AKSW/RDFUnit, http://rdfunit.aksw.org/

9 Processes, including algorithms and models, through which a knowledge graph is extracted from the unstructured or semi-structured text.

10 Source: https://fairassist.org/, accessed on November 2021.

11 Since “FAIR Evaluation Services” tool returns “server error” for COVID-KG findability, instead, the F-UJI result will be used which is closer to “FAIR Evaluation Services” tool results.
